# Advanced Clinical Usefulness of Ultrasonography for Diseases in Oral and Maxillofacial Regions

**DOI:** 10.1155/2010/639382

**Published:** 2010-04-27

**Authors:** Nao Wakasugi-Sato, Masaaki Kodama, Kou Matsuo, Noriaki Yamamoto, Masafumi Oda, Ayataka Ishikawa, Tatsurou Tanaka, Yuji Seta, Manabu Habu, Shinya Kokuryo, Hisashi Ichimiya, Ikuya Miyamoto, Shinji Kito, Shinobu Matsumoto-Takeda, Tetsuro Wakasugi, Yoshihiro Yamashita, Izumi Yoshioka, Tetsu Takahashi, Kazuhiro Tominaga, Yasuhiro Morimoto

**Affiliations:** ^1^Department of Oral Diagnostic Science, Kyushu Dental College, Kokurakita-ku, Kitakyushu, Fukuoka 803-8580, Japan; ^2^Department of Oral and Maxillofacial Surgery, Kyushu Dental College, Kokurakita-ku, Kitakyushu, Fukuoka 803-8580, Japan; ^3^Department of Bioscience, Kyushu Dental College, Kokurakita-ku, Kitakyushu, Fukuoka 803-8580, Japan; ^4^Department of Otorhinolaryngology, University of Occupational and Environmental Health, Kitakyushu, 807-8555, Japan

## Abstract

Various kinds of diseases may be found in the oral and maxillofacial regions and various modalities may be applied for their diagnosis, including intra-oral radiography, panoramic radiography, ultrasonography, computed tomography, magnetic resonance imaging, and nuclear medicine methods such as positron emission tomography. Of these modalities, ultrasound imaging is easy to use for the detection of noninvasive and soft tissue-related diseases. Doppler ultrasound images taken in the B-mode can provide vascular information associated with the morphology of soft tissues. Thus, ultrasound imaging plays an important role in confirming the diagnosis of many kinds of diseases in such oral and maxillofacial regions as the tongue, lymph nodes, salivary glands, and masticatory muscles. In the present article, we introduce three new applications of ultrasonography: guided fine-needle aspiration, measurement of tongue cancer thickness, and diagnosis of metastasis to cervical lymph nodes.

## 1. Introduction

**Figure 1:**
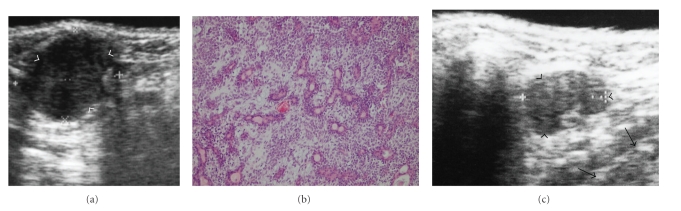
(a) B-mode in ultrasonography of a 58-year-old man with a pleomorphic adenoma in the left submandibular gland. The arrowheads indicate the mass lesion with an echogenic signal and a clear margin in the left submandibular gland. We diagnosed the mass clinically as a benign submandibular-related tumor. (b) A pathological specimen of the mass lesion in Figure 1(a). An area showing a mixture of epithelial and spindle-shaped myoepithelial elements in a variable background stroma that may be mucoid, myxoid, cartilaginous, or hyaline on the specimen. The specimen was diagnosed as a pleomorphic adenoma. (c) B-mode in ultrasonography of a 54-year-old woman with chronic lymphadenitis in the submandibular space. The arrowheads indicate the mass lesion with an echogenic signal and a clear margin in the submandibular space; the arrows indicate normal submandibular gland tissue. We clinically diagnosed the mass as chronic lymphadenitis.

**Figure 2:**
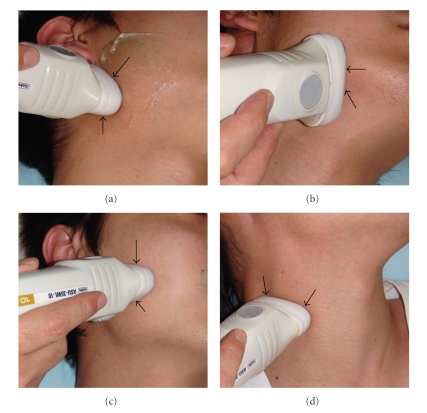
Photographs of an ultrasonographic examination of areas in the oral and maxillofacial regions. (a) The ultrasonographic probe (arrows) directly contacted the skin over the parotid gland in the coronal angle. (b) The ultrasonographic probe (arrows) directly contacted the skin over the submandibular gland in the coronal angle. (c) The ultrasonographic probe (arrows) directly contacted the skin over the masseter muscles in the axial angle. (d) The ultrasonographic probe (arrows) directly contacted the skin over the superior internal jugular vein in the axial angle.

**Figure 3:**
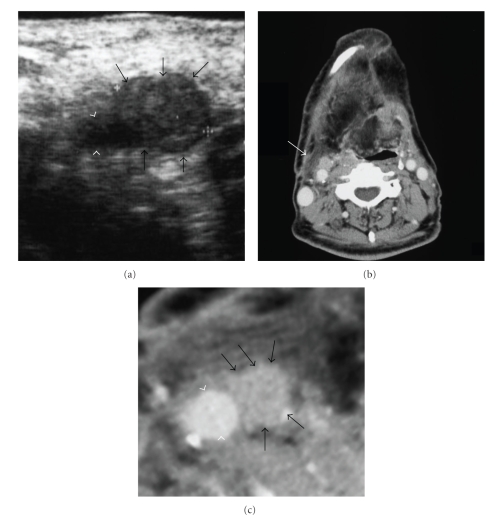
(a) B-mode in ultrasonography around the right superior internal jugular vein of a 69-year-old man with carcinoma on the right side of the tongue. The metastatic lymph node (arrows) contacts the common carotid artery (white arrowheads). (b) Computer tomography (CT) images of the same patient shown in Figure 3(a). The arrow indicates the same regions of B-mode in ultrasonography. (c) Magnification of CT images in area around arrow of Figure 3(b). The metastatic lymph node (arrows) contacts the common carotid artery (white arrowheads).

Ultrasonography (US) is easy to use for the detection of noninvasive and soft tissue-related diseases in oral and maxillofacial regions [1–4]. In ultrasound images, the B-mode shows the anatomical surface structures of soft tissues (Figures 1–3) and is commonly applied for the detection of various kinds of diseases in oral and maxillofacial regions. The ultrasound image indicates the surface structures of computed tomography (CT) and magnetic resonance (MR) images (Figure 3). Recently, Doppler ultrasound images using the Doppler effect of flow in blood vessels have also been applied to evaluate the presence or absence of vascular flow in normal tissues and in diseases of the oral and maxillofacial regions. Therefore, Doppler images associated with the B-mode can provide vascular information associated with the morphology of soft tissues [5–7]. Thus, US plays an important role in analyzing normal and abnormal anatomical structures (Figure 1). In particular, in the oral and maxillofacial regions, US may be clinically applied to evaluate salivary gland-related diseases, lymph node-related diseases, subcutaneous diseases, and tongue-related diseases [8]. However, most dentists do not know the utilities of US for the diagnosis of various kinds of oral diseases and it is very disadvantageous for patients with any of the diseases mentioned above. In the present article, therefore, we explain the significance of the clinical applications of US-guided fine-needle aspiration (FNA), ultrasound identification and measurement of tongue cancer thickness, and ultrasound-based diagnosis of metastasis to cervical lymph nodes based on the knowledge from various manuscripts acquired through the search engine “Pub-Med” (search words: ultrasonography oral and maxillofacial, ultrasonography FNA, and ultrasonography tongue).

## 2. Clinical Applications of Ultrasound Images in Fine-Needle Aspiration Biopsy

Ultrasound images using B-mode can precisely visualize normal and abnormal anatomical structures and can clearly identify the presence or absence of mass-like lesions in oral and maxillofacial regions. Therefore, US examination can readily detect and diagnose salivary gland- and lymph node-related diseases (Figure 1) and is a very useful tool for FNA biopsy (FNAB). In the B-mode of US examination of oral and maxillofacial regions, the ultrasound probe directly contacts the skin over the target examination areas at various angles, as indicated in the photographs in Figure 2.

Since Martin and Ellis first used the technique in 1930, FNAB has been clinically applied for the histologic evaluation of cervical masses [9]. FNAB is an inexpensive, rapid, and relatively accurate diagnostic method for many kinds of diseases in the oral and maxillofacial regions [10–16]. At the same time, various imaging modalities such as US, CT, and MR may also be used for the detection of lesions and for examinations that safely avoid disturbing important blood vessels and organs. Of these modalities, US imaging is the easiest to use, the least expensive, and the least invasive [12–14]. In addition, the accuracy of US-guided FNAB has been shown to be relatively high despite being noninvasive [12–16]. Al-Khafaji et al. reported that needle aspiration of parotid masses at a major referral cancer center had a sensitivity of 82%, a specificity of 86%, and an overall diagnostic accuracy of 84% using 154 parotid gland masses [14]. Studies have reported that the pathological diagnoses of lesions obtained using US-guided FNAB agreed with the final pathological diagnoses after surgical dissection in about 90% of 37 cases [15, 16]. Therefore, we applied US to guide FNAB for the diagnosis of cervical masses, such as those in metastatic lymph nodes, and salivary gland-related masses.

However, in about 10%–20% of cases, adequate pathological specimens could not be obtained. There have been few reports of major complications, but hematomas have been reported [10, 17]. We applied the B-mode of US for the detection of many kinds of blood vessels because color Doppler US was not available in our dental hospital (Figure 3). We have not yet experienced significant complications from the injury of vasculature in performing US-guided FNAB and have still detected vasculature using the B-mode of US without color Doppler sonography (Figure 3). If color Doppler US is available before a biopsy, routine use of color Doppler US has been encouraged to guide the cutting needle to areas of the lesion showing sufficient vascularity [18].

**Figure 4:**
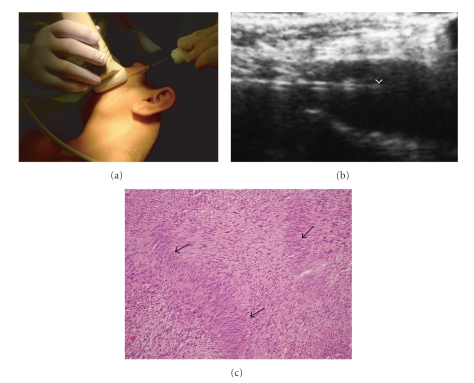
(a) View of ultrasonography-guided fine-needle aspiration biopsy including cutting-needle biopsy of the left masseter muscle in a 53-year-old woman with neurilemoma using the newly developed Monopty biopsy instrument. (b) An ultrasound image showing successful centesis (arrowhead) of the needle into the mass suspected to be a neurilemoma. (c) Antoni type A areas showing nuclear palisading (arrows) on the specimen. The specimen was diagnosed as a neurilemoma.

When performing US-guided FNAB as part of the preoperative assessment of head and neck lesions, including diagnosing lymph node metastases, we have used the newly developed Monopty biopsy instrument (MBI) (Monopty, Bard Urologic Division; Covington, GA, USA) (Figure 4(a)) [8]; few of the pathological samples obtained with this instrument had crush artifacts, injuries to the tissues caused during excision by the rushed movement of biopsy instruments, or were obscured by blood; all of which are problems that are commonly associated with manual biopsy techniques. We used the Monopty biopsy instrument to prick a mass percutaneously (Figure 4(a)) and successfully achieved centesis (arrowhead) of the needle into the mass, apparent from the ultrasound image (Figure 4(b)). The specimen in the Monopty biopsy instrument was subsequently pathologically examined and a conclusive diagnosis was reached (Figure 4(c)).

## 3. Interventional Radiology Using Fine-Needle Aspiration by Ultrasonography

We have injected OK-432 (picibanil), a biological response modifier used for sclerotherapy, into masses as nonsurgical treatment for ranulas in the oral floor [19–23]. Roh and Kim reported total or nearly total shrinkage in six of nine cases of ranulas [21]. In a followup after the last sclerotherapy, recurrence of the ranula was observed in only one patient [21]. No significant complications were observed; four patients reported fever and mild local pain lasting for 2–4 days after treatment [21]. Others reported that seven (33%) of 21 patients with plunging ranulas showed total shrinkage and resolution [22]. This technique using US seems to be effective for OK-432 administration to masses as a non-surgical treatment for ranulas in the oral floor [19–24].

**Figure 5:**
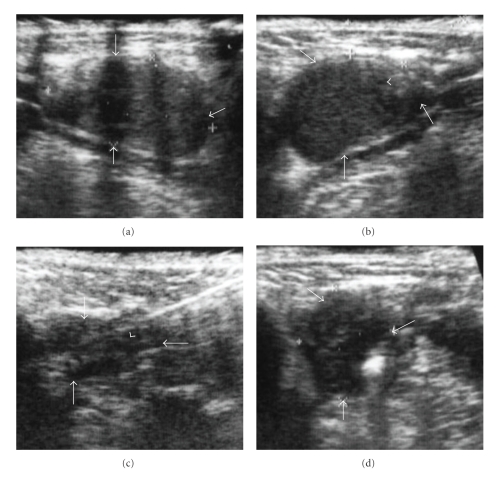
(a) An ultrasound image showing a mass in the right ranula of a 10-year-old girl. (b) An ultrasound image showing the successful penetration of a needle (arrowhead) in a syringe into the ranula (arrows). (c) An ultrasound image showing the decreasing mass (arrows) by aspiration using a syringe. The arrowhead indicates the needle. (d) An ultrasound image showing the increasing mass (arrows) by administration of OK-432 using a syringe.

In performing OK-432 treatment in our dental hospital, we apply US images both for confirmation of the appropriate removal of cystic fluid from the ranula (Figure 5(a)) and for confirmation of OK-432 administration into the ranula (Figure 5(b)). We confirm that the end of the needle penetrates the ranula and observe that the mass decreases gradually by removal of the cystic fluid from the mass (Figure 5(c)). We also confirm that the end of the needle is repositioned into the mass and observe that the mass increases gradually by the administration of OK-432 into the mass (Figure 5(d)).

## 4. Clinical Applications of Ultrasound Images in the Diagnosis of Primary Lesions of the Tongue

**Figure 6:**
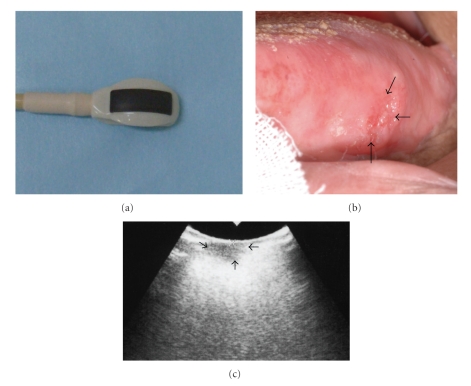
(a) View of the probe for intra-oral ultrasound examination. (b) View of the tumor in a 65-year-old man with carcinoma (arrows) on the left side of the tongue. (c) An ultrasound image showing the precise thickness of the tumor on the left side of the tongue (arrows) using intra-oral ultrasonography.

**Figure 7:**
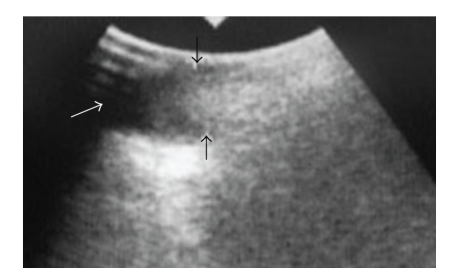
View of the imperfect examination (white arrow) using intra-oral ultrasonography because the tumor is located near the base of the tongue in a 67-year-old man with carcinoma (arrows) on the left side of the tongue.

It is very apparent that tumor thickness in oral squamous cell carcinoma of the tongue is highly related to the occurrence of cervical metastasis. Accurate preoperative assessment is indispensable to improve therapeutic effects. Particularly in cases of tongue cancer, US imaging is often used to accurately estimate tumor size or thickness and to define adequate resection margins with tumor extension and deep infiltration [25–31]. The method for estimating tongue cancer thickness involves direct contact with the tumor by a small US probe of 1 × 2.7 cm (Figure 6). In our dental hospital, intra-oral US of the tongue is typically performed with a 7.5 MHz linear array transducer of 1 × 2.7 cm (Aloka, Tokyo, Japan) (Figure 6(a)). Using this method, we have elucidated that intra-oral US offers the most exact assessment of tongue tumor thickness [29–31]. In our previous reports, we have demonstrated that the accuracy of tongue tumor thickness could be measured within 1 mm with intra-oral US [29–31]. Shintani et al. [32] showed the superiority of US over CT and MRI for its ability to measure tumor thickness within 1 mm using 24 patients and pathological specimens, and Yuen et al. [33] concluded that US was an accurate assessment modality for preoperative measurements of tumor thickness using 54 patients and pathological specimens. Additional studies have similarly reported the exact assessment of tongue tumor thickness using intra-oral US [25–33]. However, this technique using intra-oral US might provide incorrect results for the assessment of tumor thickness when the US probe cannot contact the lesion appropriately (Figure 7). In most of these cases, the tongue tumors are too large for the size of US probe (about 1 × 2 cm) and the size disparity between the tumor and the probe makes appropriate contact difficult. Both US probe and tongue tumors are convex shapes. In particular, when tongue tumors are located near the base of the tongue, the US probe cannot reach these tumors.

**Figure 8:**
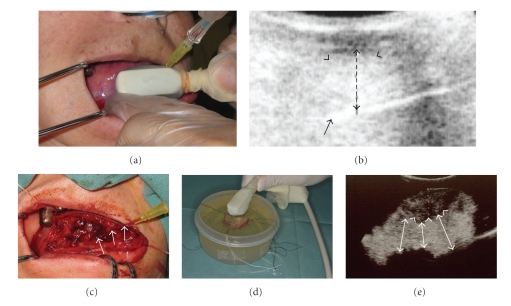
Intraoperative determination methods of tumor thickness and resection margin in tongue carcinoma using ultrasonography. (a) A view of the needle placed approximately 10 mm from the deepest portion of the tumor invasion front, with the deep surgical clearance distance verified with live ultrasound monitoring. (b) An ultrasound image of Figure 8(a). The image demonstrating the needle (arrow) placed approximately 10 mm (dotted arrows) from the deepest portion of the tumor invasion front (arrowheads). (c) Resection was performed by the use of an elastic needle (arrows) as a landmark to show the deep surgical clearance of 10 mm. (d) After solidification of the gelatin-embedded specimen, the sample including the tumor in the gelatin-embedded specimen was directly observed by US. (e) An ultrasound image of the sample in Figure 8(d) indicating the appropriate resection placed approximately 10 mm (arrows) from the deepest portion of the tumor invasion front (arrowheads).

Very recently, we developed and confirmed a method to easily allow operators to assess and confirm the surgical clearance of tongue carcinomas intraoperatively using intra-oral US (Figure 8) [29, 31]. Briefly, the tip of the needle was placed approximately 10 mm from the deepest portion of the tumor invasion front, with the deep surgical clearance distance verified with live ultrasound monitoring (Figures 8(a) and 8(b)). Resection was performed using the elastic needle as a landmark to show the deep surgical clearance of 10 mm (Figure 8(c)). Immediately after resection of the tumor with the maximum possible safety margin clearance, a fresh specimen was embedded in a gelatin solution. After solidification of the gelatin-embedded specimen within 10~20 minutes, direct US observation of the sample, including the tumor in the gelatin-embedded specimen, was performed (Figure 8(d)). Based on the imaging findings on the extent of resection around the tumor by US for the sample, we could decide whether additional resection around the tumor in the tongue was necessary (Figure 8(e)). The total time for the present technique was within 30 minutes and we could clinically apply this technique during real surgical procedures for tongue tumors. Using this new technique, we can safely, precisely, and subjectively decide the resection areas of tumors [29, 31]. If an inadequate margin is encountered in some portions, additional resection can be performed immediately during the same operation. Therefore, in a previous report of 13 cases with T1N0 (4 cases) and T2N0 (9 cases) tongue squamous cell carcinoma, we evaluated the significance of the technique using pathological specimens after resection as the gold standard [31]. Our technique showed a high degree of reliability in comparison with the histologic measurements for tumor thickness, because the mean difference between them was 1.21 mm, which indicated a good correlation and no underestimation in any case. Because there was no tumor recurrence, we speculated that there was no remnant of the tumor in the tongue after the surgical resection of the tumor in all cases [31]. In addition, in all cases, tumors had been perfectly excised based on findings on the pathological specimens after the surgical resection of the tongue tumors [31]. Kodama et al. suggested that this technique provided a definitive physical reference during resection and could be performed easily with minimal tissue distortion [31].

## 5. Clinical Applications of Ultrasonography in the Diagnosis of Metastatic Lymph Nodes in Oral Cancer

US can be used to assess lymph nodes in patients with oral cancers. Many studies have reported the usefulness of US for the diagnosis of lymph node metastases [34–39]. In these reports, ultrasound scanning had a diagnostic accuracy rate of about 90% in cervical lymph node staging [37] and US was significantly better than CT in depicting metastatic cervical nodes using 209 cervical lymph nodes from 62 patients and pathological specimens [39]. Furthermore, the accuracy rate for the diagnosis of metastatic lymph nodes ranged from 75% to 85%. Our experience has shown that it is very useful to evaluate the presence or absence of cervical lymph node metastasis of oral cancer after patients have undergone surgical treatment and/or radiotherapy. In regions that have been excised and exposed to radiation, in addition to the disappearance of the primary tumor, normal tissues are replaced with a cicatrix produced by granulation tissues. Furthermore, the cutaneous and subcutaneous tissues are difficult to palpate. Therefore, the diagnosis of metastases by direct palpation of the remaining lymph nodes in the neck becomes more difficult after cancer treatment. Thus, US is becoming increasingly more useful for detecting subclinical lymph node metastases. Doppler US evaluates the vascular pattern of nodes and helps to identify the malignant nodes [40, 41]. Normal lymph nodes have extensive vascularity originating in the hilus and branching radially towards the periphery [41, 42]. Conversely, the metastatic lymph nodes have peripheral vasculature that runs along the periphery of nodes and no vasculature around the hilus [41, 42]. We can easily distinguish the differences between the particular ultrasound findings of the two. According to some reports, after irradiation, the enhanced Doppler signals contribute to better visualization of the vessels and better detection of any vascular abnormalities based on a comparison between pathological and ultrasonographic findings [43, 44]. Thus, particular attention should be paid to followup imaging examinations of patients with oral cancers before and after radiotherapy to detect lymph node metastases [43].

**Figure 9:**
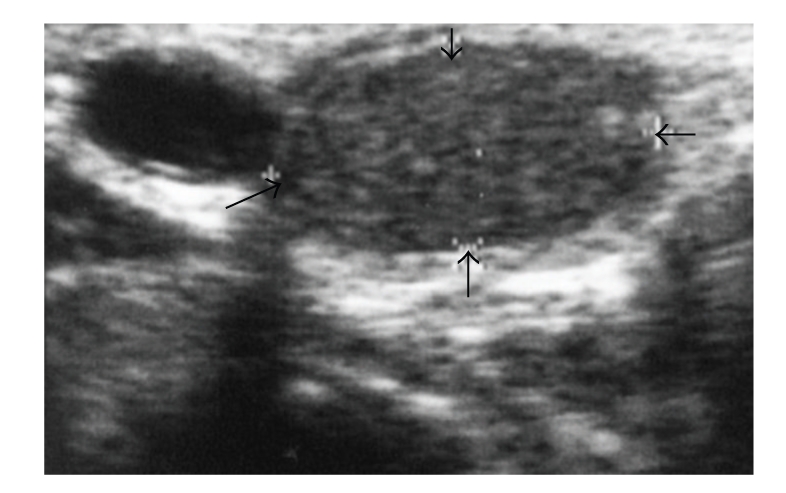
Ultrasound images in the right half of the thyroid gland of an 81-year-old man with squamous cell carcinoma on the right side of the tongue and metastasis in one of the superior internal jugular lymph nodes. A 2.6-cm echogenic mass (arrows) in the right half of the thyroid gland is shown.

When surveying the metastasis in cervical lymph nodes in patients with oral cancer, we suggested the clinical significance of additional ultrasonographic examination for thyroid glands [45]. In that report, we elucidated that over 30% of patients with oral squamous cell carcinoma have a relatively high rate of abnormal findings in the thyroid gland that can be detected by US. In addition, as subject age increased, the rate of detection of abnormal thyroid gland findings on US significantly increased; this increase was particularly prominent for men. In one case, a 2.3-cm echogenic mass in the right side of the thyroid gland was detected in a patient with a lesion on the right side of the tongue that was diagnosed as squamous cell carcinoma by biopsy in another hospital and one metastatic lymph node was also determined (Figure 9). Moreover, particular attention should be paid to thyroid gland abnormalities if patients had oral squamous cell carcinoma on the floor of the mouth or in the maxillary gingiva. Moreover, a relative high rate of patients showed enlargement in the size of the lesion upon followup examination with US. Therefore, when such findings appeared during followup examinations, we promptly instruct patients to consult specialists to further search for lesions in the thyroid gland.

In our other study, we recommended 4–6 times per neck as one standard axial scanning period for the survey of cervical lymph nodes including the thyroid gland by US in patients with oral squamous cell carcinoma (Wakasugi-Sato et al. (submitted for publication)). In addition, beginning users of US for the detection of cervical lymph nodes should take care not to overlook accessory spinal lymph nodes.

## 6. Conclusions

In the present review, we described the clinical application of ultrasonography (US) for the diagnosis of various diseases in oral and maxillofacial regions, including the introduction of new trials of US such as FNA using US, the decision of surgical margins of tongue cancer lesions using US, and the clinical necessity of examination for thyroid gland-related diseases when surveying the diagnosis of cervical metastasis of lymph nodes by US.

US is easy to use for the noninvasive detection of soft tissue-related diseases in oral and maxillofacial regions. Therefore, B-mode using a relative large probe with 7.5~10 MHz should be preferentially selected for the differential diagnosis of soft tissue surfaces including salivary gland- and lymph node-related diseases. Conversely, B-mode using a small probe with 7.5~10 MHz should be applied to determine the presence or absence of tongue mass-like lesions, including benign or malignant tumors, of the tongue. In addition, the modality is very significant for the decision of surgical margins of tongue cancers. Doppler mode in US is a very useful modality in the differential diagnosis between normal and metastatic lymph nodes in patients with oral squamous cell carcinoma. FNA for mass-like lesions and OK-432 administration for ranulas also began as interventional radiology techniques using US in the oral and maxillofacial regions. Further investigation is needed to standardize the methods of US for diagnosing various kinds of diseases in the oral and maxillofacial regions. The clinical application of US in the oral and maxillofacial regions should be advocated in various publications.
